# Types of Challenges and Barriers in Accessing Paediatric Palliative Care in Romania: A Qualitative Study Based on Focus Groups Guided by a Semi-Structured Discussion Guide

**DOI:** 10.3390/medicina62010057

**Published:** 2025-12-28

**Authors:** Mihaela Hizanu Dumitrache, Liviu Stafie, Alina Plesea-Condratovici, Dana Elena Mindru, Camer Salim, Eva Maria Elkan, Mădălina Duceac Covrig, Mădălina Nicoleta Matei, Ciprian Adrian Dinu, Letiția Doina Duceac

**Affiliations:** 1 Faculty of Medicine and Pharmacy, Doctoral School of Biomedical Sciences, “Dunărea de Jos” University of Galați, 47 Domnească Street, 800008 Galați, Romania; mh202@student.ugal.ro (M.H.D.); madalina.duceac@ugal.ro (M.D.C.); 2 Faculty of Medicine, “Grigore T. Popa” University of Medicine and Pharmacy, 700115 Iasi, Romania; liviu.stafie@umfiasi.ro (L.S.); mindru.dana@umfiasi.ro (D.E.M.); 3 Faculty of Medicine and Pharmacy, “Dunărea de Jos” University of Galați, 47 Domnească Street, 800008 Galați, Romania; cojocarumariaeva@yahoo.com (E.M.E.); madalina.matei@gmail.com (M.N.M.); cdinu@ugal.ro (C.A.D.); letimedr@yahoo.com (L.D.D.); 4 Faculty of Medicine, Ovidius University of Constanta, 1 Universității Street, Campus-Corp A, Etaj 1, 900470 Constanța, Romania

**Keywords:** palliative care, paediatrics, life-limiting illnesses, complex needs, perception, recovery, access to services

## Abstract

*Background and Objectives:* Paediatric palliative care in Romania is underdeveloped and unevenly distributed, which creates major difficulties in accessing services for children with life-limiting illnesses and their families. The lack of a dedicated national strategy, the shortage of specialised staff, and socio-economic barriers exacerbate the vulnerability of these groups. This study aimed to explore parents’ and caregivers’ experiences, to analyse the perspectives of public institutions and NGOs involved in supporting these children, and to identify the main barriers and facilitators in accessing paediatric palliative care. *Materials and Methods:* Given that all data were collected exclusively through focus group discussions, this study employed a qualitative design based on three focus groups guided by a semi-structured interview guide. The analysis was conducted using MAXQDA software, which enabled the coding and synthesis of emerging themes. Participants were parents/caregivers of children with life-limiting illnesses, representatives of public institutions, and members of relevant NGOs in Bacău County, Romania. Purposive sampling was used to capture diverse and experience-based perspectives, resulting in a total of 24 participants across three focus groups—parents and caregivers (*n* = 11), public institution representatives (*n* = 7), and NGO representatives (*n* = 6). No individual semi-structured interviews were conducted. *Results:* The analysis highlighted a complex typology of medical, emotional, social, educational, and spiritual needs of children and their families. Parents reported experiences of abandonment in the curative system, emphasising the importance of pain control, safety, and community support. Public institutions acknowledged the lack of skills and inter-sectoral coordination, while NGOs pointed to structural barriers and the low visibility of these children. Major needs include access to specialised care, psychological support, respite services for carers, financial and administrative assistance, education, and spiritual counselling. A significant obstacle is the lack of up-to-date statistical data needed to inform public policy. *Conclusions:* Paediatric palliative care should be considered a national priority through the development of a dedicated strategy, the expansion of specialised services, and the strengthening of partnerships between the public and non-governmental sectors. An integrated, child- and family-centred approach addressing the medical, social, emotional, and spiritual dimensions of care is essential. The results highlight the need for continuous staff training, information campaigns, and community support mechanisms to reduce inequalities and improve the quality of life of children with life-limiting illnesses.

## 1. Introduction

Paediatric palliative care is an integral component of health systems, aiming to improve the quality of life of children with incurable, life-limiting, or life-threatening conditions through comprehensive medical, psychological, social, and spiritual support [1]. Despite its recognised importance, access to paediatric palliative care remains limited worldwide. The World Health Organisation estimates that approximately 21.5 million children require palliative care globally; however, only a small proportion receive appropriate services, particularly in low- and middle-income countries [2,3].

In Romania, the provision of paediatric palliative care is insufficient and unevenly distributed. In 2019, only 50 beds were dedicated to paediatric palliative care nationwide, indicating a substantial gap between service availability and actual needs [4]. More recent data (2022–2023) identified 14,499 children who would require palliative care services, with marked disparities across counties, age groups, diagnostic categories, and place of residence [5]. A significant proportion of affected children live in rural areas, where access to specialised care is further limited by geographical distance, financial constraints, and low levels of medical information [5].

According to the World Health Organisation classification, conditions requiring paediatric palliative care include life-threatening diseases with uncertain outcomes, life-limiting conditions with no reasonable prospect of cure, progressive incurable disorders, and non-progressive conditions associated with severe disability and increased risk of complications [6,7]. Although distinctions can be made between terminal, life-limiting, and life-threatening illnesses [8,9,10], all involve complex care trajectories and long-term support needs for both children and their families.

International evaluations continue to place Romania among countries with isolated paediatric palliative care services, characterised by a limited number of specialised centres, most of which are supported by non-governmental organisations [11]. The lack of a coherent national strategy, shortages of trained healthcare professionals, limited funding, and pronounced regional inequalities represent persistent systemic barriers to equitable access [11]. While the need to expand and integrate paediatric palliative care services has been acknowledged, there is limited empirical evidence capturing the perspectives of key stakeholders involved in care provision and policy implementation.

This study is informed by the biopsychosocial model, which conceptualises child health and caregiving as the result of interacting medical, psychological, social, and spiritual factors [12,13], and by Bronfenbrenner’s ecological systems theory, which situates these experiences within broader family, community, institutional, and policy contexts [14]. Together, these frameworks support a multidimensional analysis of needs, barriers, and enabling factors in paediatric palliative care.

Against this background, the present study aims to explore the experiences and perceptions of parents caring for children with life-limiting illnesses, representatives of public institutions, and non-governmental organisations involved in paediatric palliative care in Romania; to identify perceived needs, challenges, barriers, and facilitating factors; to compare perspectives across stakeholder groups; and to formulate recommendations for improving paediatric palliative care services.

Accordingly, the main research question guiding the study is: What types of needs, challenges, and barriers do parents/caregivers, public institution representatives, and NGO representatives in Romania perceive in accessing paediatric palliative care for children with life-limiting conditions?

## 2. Materials and Methods

### 2.1. Study Design

This study adopted a qualitative exploratory design, using a thematic analysis approach to examine the experiences, perceptions, and perceived needs of key stakeholders involved in paediatric palliative care in Romania. Data were collected through focus group discussions guided by a semi-structured interview guide. The guide was developed based on predefined thematic areas relevant to paediatric palliative care, including medical care; psycho-emotional support; social and educational needs; access to information; respite care; spiritual concerns; and systemic issues related to service organisation, data availability, and policy development.

The focus group methodology was selected to facilitate interaction among participants, encourage the sharing of experiences, and enable the co-construction of meaning through group dialogue. This approach was considered appropriate for exploring sensitive topics, capturing collective perspectives, and identifying both shared experiences and divergent viewpoints across participant groups.

### 2.2. Setting and Participants

The study was conducted in Bacău County, Romania, between February 2024 and October 2024. A purposive sampling strategy was employed to ensure the inclusion of participants with relevant and diverse experiences related to paediatric palliative care. Eligible participants were adults directly involved in caregiving, service provision, or institutional decision making for children with life-limiting conditions.

Three focus groups were organised, each comprising a distinct stakeholder category. The first group included parents and primary caregivers of children diagnosed with life-limiting illnesses; the second group consisted of representatives of public institutions involved in healthcare, social services, or administrative processes related to child disability and chronic illness; and the third group included representatives of non-governmental organisations providing support services to children with disabilities and their families. Participants were recruited through institutional gatekeepers from paediatric palliative care centres, public health and health insurance authorities, social assistance directorates, and relevant non-governmental organisations.

Inclusion criteria required participants to be at least 18 years of age, to have direct caregiving responsibilities or professional involvement in paediatric chronic or palliative care, to be able to provide informed consent, and to be fluent in Romanian. Individuals were excluded if they were unable to participate in group discussions due to cognitive or emotional impairment or if they were experiencing an acute psychological crisis at the time of recruitment. The final sample comprised predominantly women, reflecting the gender distribution commonly observed in caregiving roles and social and health service professions.

### 2.3. Data Collection Procedures

Data were collected exclusively through focus group discussions; no individual interviews were conducted. Each focus group was moderated by a trained researcher and followed the same semi-structured discussion guide to ensure consistency across groups while allowing participants to introduce additional topics considered relevant. Discussions explored participants’ experiences with medical care and symptom management, emotional and psychological support, social and financial challenges, communication with professionals, educational and informational needs, access to respite care, spiritual or existential concerns, and perceived structural or systemic barriers to paediatric palliative care.

Each focus group session lasted between 90 and 120 minutes. All discussions were audio-recorded with the participants’ consent and subsequently transcribed verbatim for analysis.

### 2.4. Data Analysis

A multi-stage qualitative analysis was performed using MAXQDA Analytics Pro (2024) software. Verbatim transcripts of all focus group discussions were first prepared and then imported into the software for coding. An initial deductive coding phase was conducted based on the predefined thematic framework underpinning the study. This was followed by an inductive coding process to capture emergent themes arising directly from participants’ narratives. Related codes were subsequently grouped into broader themes and subthemes, allowing for the development of coherent thematic structures.

Constant comparison was applied across the three participant categories to identify areas of convergence and divergence in perspectives. The resulting thematic structures were reviewed and validated by the research team to enhance analytic rigor, consistency, and interpretive credibility. This combined inductive–deductive approach enabled a systematic analysis informed by both theoretical considerations and participants’ lived experiences.

### 2.5. Ethical Considerations

The study was conducted in accordance with the principles of the Declaration of Helsinki and received approval from the Ethics Committee of the Lumina Association—Children’s Palliative Care Centre Bacău (Decision No. 02/04.01.2024). Ethical approval covered all stages of the research process, including participant recruitment, data collection, data management, and data protection procedures.

All participants received detailed information about the study objectives, procedures, confidentiality safeguards, and their right to withdraw at any time without consequence, and they provided written informed consent prior to participation. To ensure confidentiality, personal identifiers were removed from transcripts and replaced with anonymised codes in all analyses and reports. Audio recordings and transcripts were stored on encrypted devices accessible only to the research team and will be securely destroyed after completion of the analyses. Given the sensitive nature of the topics discussed, moderators applied trauma-informed communication strategies and allowed participants to pause or decline to answer questions if needed. The study was designed to ensure non-stigmatising reporting and to generate findings relevant to the improvement of paediatric palliative care services in Romania.

## 3. Results—Typology of Identified Problems and Needs

A total of 24 participants were included in this study. Recruitment took place in Romania through purposive sampling, facilitated by institutional gatekeepers such as a paediatric palliative care centre, the County General Directorate for Social Assistance and Child Protection (DGASPC), Health of Insurance House, Public Health Directorate, and several local NGOs working with children with disabilities or chronic conditions.

Participants represented three key stakeholder groups:

- Parents/caregivers (*n* = 11) of children diagnosed with life-limiting conditions (neurological, genetic, oncological or multisystemic disorders);
- Public institution representatives (*n* = 7), including physicians, psychologists, nurses, and social workers;
- NGO representatives (*n* = 6), mainly from organisations providing disability support, volunteer services, and community-based assistance.

The gender distribution was predominantly female (22 women and 2 men), reflecting gender patterns typically observed in caregiving and social sectors.

Building on the perspectives of all participant groups, the qualitative analysis identified one central overarching theme—Typology of Needs of Children with Life-Limiting Conditions and Their Families—encompassing eight interrelated categories: medical, psycho-emotional, social, educational, informational, respite, spiritual, and policy-related needs. All categories were reported across stakeholder groups, although their emphasis varied according to participants’ roles and experiences (Table 1).

### 3.1. Medical Needs

Medical needs were described by parents and caregivers as the most immediate and pressing concern, particularly in interactions with curative healthcare services. Several participants recalled encounters marked by limited therapeutic options and minimal guidance, which were perceived as abandonment: “There is no treatment… go home, he will die.” “They sent us back without offering any solution.”

In contrast, families associated specialised paediatric palliative care services with improved symptom control and professional responsiveness. Parents described a noticeable reduction in suffering and a clearer understanding of their child’s condition: “He was no longer in pain… he finally received the right treatment.” “For the first time, we felt someone understood his condition.”

Continuity of care emerged as an important aspect of medical support. Before referral to palliative services, parents reported repeated emergency visits: “Every two days we were in the emergency room.” Following enrolment in palliative care, families described increased stability and reassurance: “We knew we could call anytime… that gave us peace of mind.”

Representatives of public institutions acknowledged the limited capacity of curative medicine to address complex paediatric cases: “Curative treatment only goes so far.” They also highlighted gaps in professional preparedness: “Medical staff are not prepared to cope.”

NGO representatives framed medical needs within broader structural constraints, including insufficient funding and limited institutional prioritisation: “The Romanian healthcare system is underfunded and overburdened.” “They are not a priority for the authorities.”

### 3.2. Psycho-Emotional Needs

Parents and caregivers described sustained emotional strain associated with long-term caregiving, coupled with restricted access to psychological support for themselves, their children, and siblings: “We all need emotional support… not just the child. The brothers and sisters suffer too.” “There are costs we simply cannot afford when the child needs treatment first.”

Interactions with specialised teams were perceived as emotionally supportive and non-judgmental: “Here I feel listened to… without being judged.” Parents also valued opportunities to connect with peers facing similar challenges: “Meeting other parents helped me so much… I no longer felt alone.”

Public institution representatives highlighted the vulnerability of primary caregivers, most often mothers: “The mother is the one who takes everything upon herself.” They also noted emotional risks for siblings: “Siblings often feel abandoned.”

NGO representatives emphasised widespread emotional exhaustion among families and limited informal support: “Parents reach a level of emotional exhaustion.”

“Sometimes they simply need a shoulder to cry on.”

### 3.3. Social Needs

Participants described social difficulties related to administrative procedures, financial strain, and social isolation. Parents reported navigating complex bureaucratic processes without adequate guidance: “So much paperwork and no one tells you where to get it.” Ongoing care-related expenses were frequently mentioned: “We need money to go to the doctors, to buy special food for the child.”

Several parents described disrupted family relationships and social withdrawal: “My husband left… I was left alone with a sick child.”

Public institution representatives acknowledged limited capacity to address social needs within healthcare settings: “On the social side, we cannot help them.” They also described fragmented institutional responsibilities: “Go to the town hall or child protection services.”

NGO representatives pointed to shortages in social services and the complexity of families’ situations: “There are not enough social workers.” “The issues faced by all people in need are vast.”

### 3.4. Educational Needs

Parents reported receiving insufficient explanations regarding diagnosis, prognosis, and care pathways, leading to uncertainty and confusion: “They didn’t tell me anything practical.” “No one explained the diagnosis properly.”

Many expressed the need for practical guidance in daily caregiving: “I kept asking: What can I do? How can I help?”

Public institution representatives noted limited awareness among both parents and professionals: “Parents do not know when or how to reach specialists.”

“There is not enough knowledge in paediatric palliative care.”

NGO representatives emphasised the importance of early and public education: “Parents need information from the very beginning.” “People simply don’t know what palliative care means.”

### 3.5. Information Needs

Parents frequently reported that information about palliative care services was obtained through informal networks: “I found out from another parent, not from any doctor.”

Some families described discovering services late and incidentally: “We discovered the centre by chance.”

Public institution representatives highlighted persistent misconceptions: “Some professionals confuse palliative care with services for the elderly.” They also noted unclear responsibility for information provision: “Everyone assumes someone else will explain.”

NGO representatives identified fear and misunderstanding as barriers to accessing care: “Parents often think palliative care means the end.”

### 3.6. Respite Needs

Parents consistently reported the absence of respite options, resulting in continuous caregiving responsibilities: “I had no one to leave the child with—even for an hour.” This situation affected caregivers’ health and family balance: “I haven’t been for a check-up in a long time.” “I couldn’t take the other child on holiday.” Participants explicitly requested specialised and trustworthy respite services: “We need a place where we know the child is safe.”

### 3.7. Spiritual Needs

Parents referred to spiritual concerns mainly through existential questions, emotional expression, and faith-based interpretations of their child’s illness. Several parents questioned the reasons behind the illness: “I don’t know… why he hit me… he was a normal child until recently.” “Why did this happen to me and my child?”

Spirituality was also associated with the need to express grief and emotional pain: “I need a shoulder to cry on… to mourn.” Some parents linked the illness to personal guilt or moral responsibility: “To talk about all my mistakes… maybe that’s why my child is like this.”

### 3.8. Lack of Statistical Data and Implications for Public Policy

Public institution representatives reported significant limitations related to data collection and availability in paediatric palliative care. Two recurring issues were identified: outdated reporting tools and the lack of reliable data to support planning, funding, and accreditation processes.

Participants noted that data are collected using obsolete instruments: “We collect data using old forms that are no longer valid and have not been updated.”

The absence of consistent and up-to-date data was described as a barrier to policy development and resource allocation: “Any national or county strategy, access to funds… must be based on a needs analysis.” “This type of data does not exist and sometimes becomes a problem even in the accreditation process.”

## 4. Discussion

The findings of the present study are consistent with evidence reported at both European and global levels, which documents the uneven development and limited availability of paediatric palliative care services across healthcare systems. International mappings of paediatric palliative care provision reveal persistent gaps in service capacity and coverage, highlighting the need for coordinated national strategies that strengthen both hospital-based and community or home-care models [15]. In this context, the results of the present study reinforce the necessity of a coherent national framework that recognises paediatric palliative care as an integral component of healthcare systems rather than a marginal or episodic intervention.

The study findings support an understanding of paediatric palliative care as a continuous and complementary component of care, integrated alongside curative treatment rather than introduced only when curative options are exhausted. Participants emphasised the importance of coordinated, long-term support in ensuring continuity of care, appropriate symptom management, and emotional stability for children with life-limiting conditions and their families. These observations are aligned with international recommendations advocating for earlier and systematic integration of paediatric palliative care, particularly in complex care trajectories characterised by disease progression, repeated clinical deterioration, and increasing care needs.

The thematic analysis further demonstrates how organisational and structural limitations within healthcare systems are reflected in families’ lived experiences. Parents’ accounts of emotional shock, uncertainty, and erosion of trust in the healthcare system appear closely linked to insufficient consultation time, limited access to specialised expertise, and fragmented communication within curative services. Similar patterns have been reported in European and international contexts, where inconsistent development of paediatric palliative care services continues to generate disparities in access and quality of care [15]. Barriers identified in the present study including lack of clear information, administrative complexity, negative or hesitant attitudes toward palliative care among professionals, and pronounced regional inequalities are consistent with previous research and help explain delayed referrals and unequal access to services [16].

The literature highlights the central role of professional training and standardisation in improving the quality and consistency of paediatric palliative care. International frameworks proposing standards of practice provide guidance for developing quality indicators, accreditation mechanisms, and structured training curricula, contributing to greater professional competence and increased parental confidence in care delivery [17]. At the same time, research emphasises that children with life-limiting conditions continue to seek engagement in daily activities and meaningful participation in family and social life, underscoring the importance of care approaches that promote normalcy and quality of life alongside medical management [18]. In this regard, individualised care plans represent a key element of child- and family-centred care, supporting shared decision making and adaptive responses to evolving clinical and psychosocial needs [19].

Parents’ experiences described in community and home-care contexts reveal persistent challenges related to poor coordination between hospital and community services, limited flexibility of care pathways, and insufficient family-centred approaches [20]. These gaps are particularly critical given the fragile clinical trajectories of many children with life-limiting illnesses, who often experience recurrent episodes of deterioration requiring acute medical intervention or hospitalisation. Repeated hospital admissions and prolonged exposure to institutional care environments may increase clinical vulnerability and strain families’ coping resources, highlighting the importance of preventive, coordinated, and holistic care strategies that aim to stabilise the child’s condition and reduce avoidable acute care utilisation [21].

In addition, progressive complications associated with life-limiting conditions such as feeding difficulties, nutritional challenges, and other symptom-related burdens can significantly affect comfort, functional status, and overall quality of life. Addressing these issues requires timely, coordinated interventions delivered by multidisciplinary teams capable of integrating medical, nutritional, and supportive care components within a comprehensive palliative approach [22]. The emphasis placed by participants on symptom control and continuity of care in the present study provides a clear experiential basis for these clinical considerations.

Beyond medical aspects, prolonged illness trajectories and repeated hospitalisations have substantial psychosocial consequences for children and their families. Existing evidence consistently highlights the need for paediatric palliative care models that integrate continuous emotional support, family presence, and interventions aimed at preserving children’s sense of safety, dignity, and identity [23]. Parents often describe preparation for the end of a child’s life as a dynamic process involving anticipatory planning, emotional adjustment, and active participation in care decisions, reinforcing the importance of transparent communication and sustained family involvement throughout the illness trajectory [24]. From the professionals’ perspective, paediatric palliative care is recognised as an emotionally demanding and complex field requiring interdisciplinary collaboration, ongoing training, and institutional support to address both technical and emotional challenges, particularly in advanced or terminal stages of illness [25,26].

Within the Romanian context, the challenges identified in this study closely mirror findings from previous national research, which has documented underfunding, shortages of specialised personnel, limited service availability, and insufficient visibility of the needs of children with life-limiting conditions within public policy agendas [27]. Quantitative data further confirm significant territorial and demographic disparities, with a high proportion of affected children residing in rural areas, where access barriers are more pronounced [5]. The convergence of qualitative findings from the present study with existing quantitative evidence strengthens the argument for expanding specialised paediatric palliative care beds, developing regional centres, implementing mobile care teams, and investing in targeted education and training programmes.

The results also have important implications for clinical practice and social policy. From a healthcare perspective, paediatric palliative care should be prioritised as an integral element of treatment planning and supported by continuous professional education and multidisciplinary service models capable of addressing medical, emotional, social, and spiritual needs simultaneously. At the social policy level, excessive bureaucracy, poverty, limited community support, and persistent stigmatisation exacerbate families’ vulnerability and call for coherent public policies aimed at reducing inequalities in access to care. Evidence suggests that expanding respite care services can reduce caregiver psychological distress and prevent burnout, thereby contributing to family stability and sustainability of care [28]. Strengthened collaboration between public institutions and non-governmental organisations is therefore essential to ensure comprehensive and accessible paediatric palliative care.

## 5. Conclusions

This qualitative study provides an in-depth examination of the needs, challenges, and barriers perceived by parents and caregivers, public institution representatives, and non-governmental organisation representatives involved in paediatric palliative care in Romania. The findings highlight that children with life-limiting conditions and their families face complex and interrelated medical, psycho-emotional, social, educational, and informational needs that cannot be adequately addressed through fragmented or exclusively curative approaches.

This study makes a scientific contribution by offering original qualitative evidence in a field that remains insufficiently explored at the national level and by complementing existing quantitative data. By capturing experiential, emotional, psychosocial, and spiritual dimensions of care, the findings provide insights that are not accessible through epidemiological analyses alone and underscore the value of integrating qualitative and quantitative evidence in service development and policy planning.

From a clinical perspective, the results emphasise the importance of integrating paediatric palliative care as a continuous component of care trajectories for children with life-limiting conditions. Multidisciplinary service models, supported by ongoing professional training, are essential to ensure continuity of care, effective symptom management, and comprehensive support for both children and their families.

At the level of public policy and social services, the findings draw attention to persistent structural barriers, including excessive bureaucracy, territorial inequalities, limited availability of specialised services, and insufficient coordination between healthcare and social protection systems. Addressing these challenges requires coherent public policies focused on equity, the expansion of specialised and community-based services, and strengthened collaboration between public institutions and non-governmental organisations. The development of respite care services represents a particularly important intervention to support caregiver wellbeing and family stability.

Several limitations should be acknowledged. The qualitative design and the geographically limited sample restrict the generalisability of the findings at the national level, and participants’ perspectives may reflect context-specific experiences. Nevertheless, the consistency of the results with existing national and international evidence supports their relevance and transferability.

Overall, the findings underline the need for a comprehensive, child- and family-centred approach to paediatric palliative care in Romania, grounded in coordinated service provision, informed public policies, and sustained investment in professional education and community awareness. These conclusions may inform future research, guide service development, and support the formulation of national strategies aimed at improving access to and quality of paediatric palliative care.

## Figures and Tables

**Table 1:** Summary of major themes, participant categories, and illustrative quotes.

Major Theme	Parents/Caregivers—Key Findings and Quote	Public Institutions—Key Findings and Quote	NGO Representatives—Key Findings and Quote
Medical needs	Felt abandoned by curative care; relief in specialised palliative services. “There is no treatment… go home.”	Curative model insufficient; lack of specialised skills. “Curative treatment only goes so far.”	Underfunded system; low visibility of children with rare diseases. “The system is underfunded and overburdened.”
2. Psycho-emotional needs	Emotional overload; need for counselling; value of trust. “Here I feel listened to.”	High maternal vulnerability; sibling exclusion risk. “Siblings often feel abandoned.”	Parental exhaustion; stabilising effect of palliative teams. “Parents reach a level of emotional exhaustion.”
3. Social needs	Bureaucracy; financial hardship; isolation. “So many doors to open… and no one answers.”	Fragmented responsibilities; limited social support in hospitals. “On the social side, we cannot help them.”	Shortage of social workers; multidimensional vulnerability. “There are not enough social workers.”
4. Educational needs	Lack of clear diagnosis explanation; confusion about care pathways. “We were moved from one hospital to another… without explanation.”	Need for parental guidance; professional training gaps. “Parents don’t know the medical pathway.”	Need for community campaigns and school-based education. “People don’t know what palliative care means.”
5. Information needs	Learn about services by chance; late access. “I found out from another parent.”	Misconceptions about palliative care; unclear institutional roles. “Even specialists confuse it with elderly care.”	Emotional barriers and stigma. “Parents think palliative care means the end.”
6. Respite needs	No alternative caregiver; self-neglect; impact on siblings. “I had no one to leave the child with.”	Essential for maternal wellbeing; must be specialised. “Especially the mother needs a break.”	Urgent need for safe respite centres. “They need a place to leave their child safely.”
7. Spiritual needs	Existential questioning and guilt. “Why did this happen to my child?”	—	—
8. Need for data and policy	—	Outdated reporting tools; lack of epidemiological data. “We collect data using old forms.”	—

## Data Availability

All data from the first author and the corresponding author are available on request.

## Abbreviations

Abbreviation	Explanation
WHO	World Health Organisation
NGO	Non-governmental organisation
MAXQDA	Qualitative analysis software
DGASPC	General Directorate for Social Assistance and Child Protection
CAS	Health Insurance House
DSP	Public Health Directorate
